# One-Pot *De Novo* Synthesis of [4Fe-4S] Proteins Using a Recombinant SUF System under Aerobic Conditions

**DOI:** 10.1021/acssynbio.3c00155

**Published:** 2023-07-19

**Authors:** Po-Hsiang Wang, Shota Nishikawa, Shawn Erin McGlynn, Kosuke Fujishima

**Affiliations:** † Department of Chemical Engineering and Materials Engineering, National Central University, Taoyuan 32001, Taiwan; ‡ Graduate Institute of Environmental Engineering, National Central University, Taoyuan 32001, Taiwan; § Earth-Life Science Institute, Tokyo Institute of Technology, Tokyo 152-8550, Japan; ∥ School of Life Science and Technology, Tokyo Institute of Technology, Tokyo 152-8550, Japan; ⊥ Blue Marble Space Institute of Science, Seattle, Washington 98154, United States; # Graduate School of Media and Governance, Keio University, Fujisawa 252-0882, Japan

**Keywords:** Fe−S cluster, reconstituted cell-free protein synthesis, aconitase, cofactor regeneration, SUF helper protein, redox enzymes

## Abstract

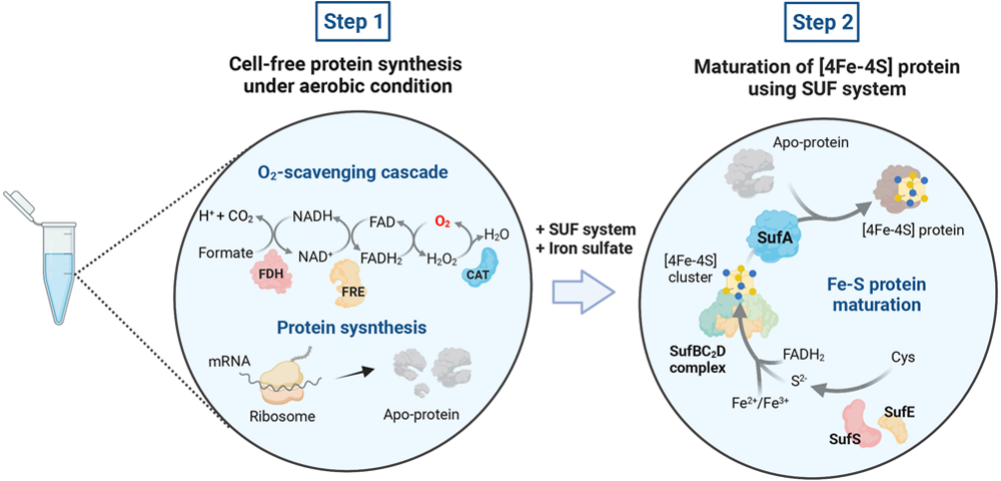

Fe–S clusters are essential cofactors mediating electron transfer in respiratory and metabolic networks. However, obtaining active [4Fe-4S] proteins with heterologous expression is challenging due to (i) the requirements for [4Fe-4S] cluster assembly, (ii) the O_2_ lability of [4Fe-4S] clusters, and (iii) copurification of undesired proteins (e.g., ferredoxins). Here, we established a facile and efficient protocol to express mature [4Fe-4S] proteins in the PURE system under aerobic conditions. An enzyme aconitase and thermophilic ferredoxin were selected as model [4Fe-4S] proteins for functional verification. We first reconstituted the SUF system *in vitro via* a stepwise manner using the recombinant SUF subunits (SufABCDSE) individually purified from *E. coli*. Later, the incorporation of recombinant SUF helper proteins into the PURE system enabled mRNA translation-coupled [4Fe-4S] cluster assembly under the O_2_-depleted conditions. To overcome the O_2_ lability of [4Fe-4S] Fe–S clusters, an O_2_-scavenging enzyme cascade was incorporated, which begins with formate oxidation by formate dehydrogenase for NADH regeneration. Later, NADH is consumed by flavin reductase for FADH_2_ regeneration. Finally, bifunctional flavin reductase, along with catalase, removes O_2_ from the reaction while supplying FADH_2_ to the SufBC_2_D complex. These amendments enabled a one-pot, two-step synthesis of mature [4Fe-4S] proteins under aerobic conditions, yielding holo-aconitase with a maximum concentration of ∼0.15 mg/mL. This renovated system greatly expands the potential of the PURE system, paving the way for the future reconstruction of redox-active synthetic cells and enhanced cell-free biocatalysis.

## Introduction

**Figure 1:**
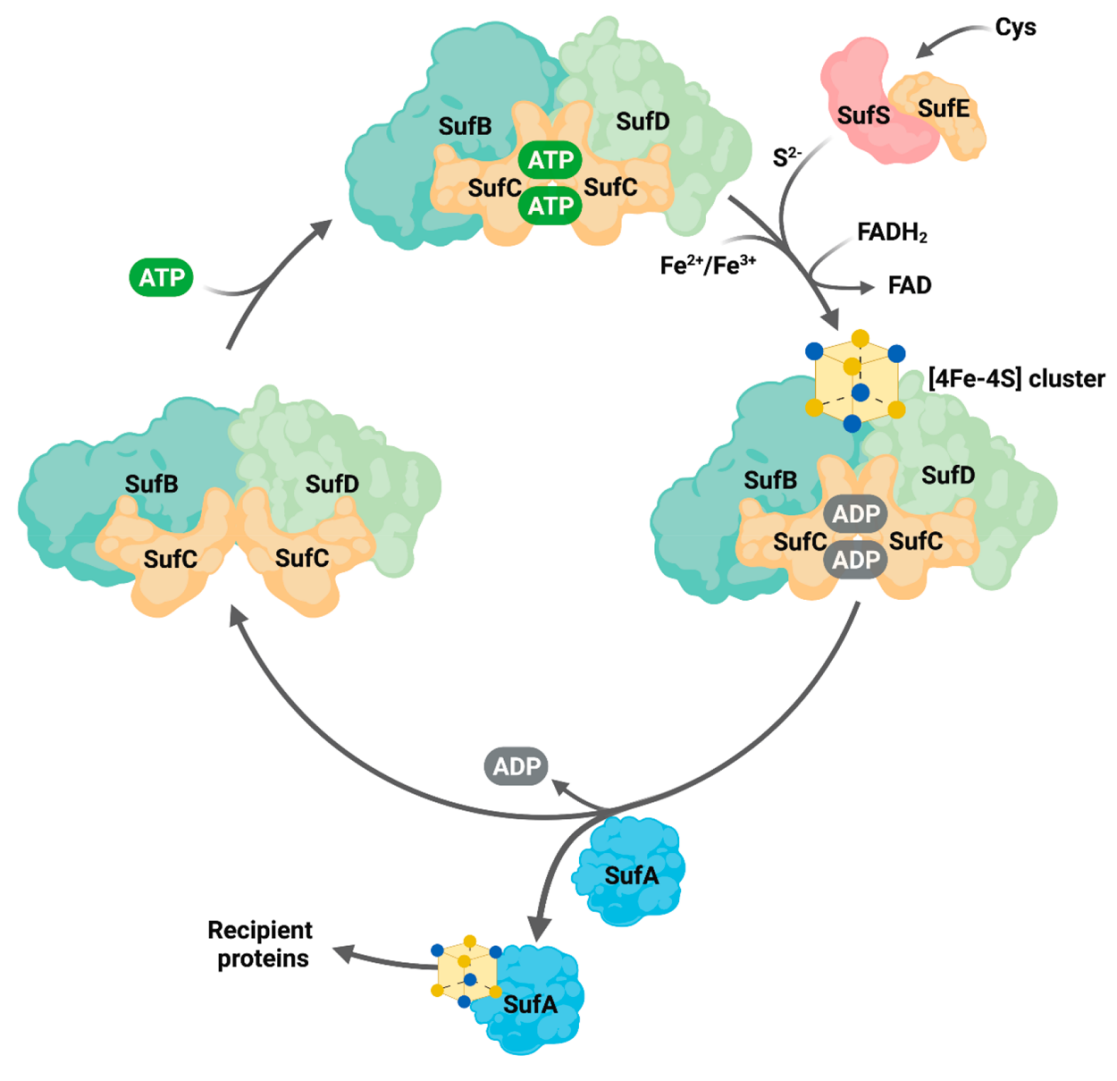
Schematic diagram of the SUF pathway. The SufBC_2_D complex acts as a scaffold, receiving the sulfide (S^2–^) group from the SufES complex and incorporating Fe^2+^ and/or Fe^3+^ depending on the type of cluster for Fe–S cluster assembly.^19^ FADH_2_ is used for Fe^3+^ reduction and [4Fe-4S] cluster assembly is driven by ATP hydrolysis. The assembled [4Fe-4S] cluster is then transferred to the SufA, which further transfers the cluster to the recipient proteins.

Iron–sulfur (Fe–S) clusters are essential prosthetic groups of many redox-active proteins and are employed by organisms across all three domains of life, serving as electron shuttles in a variety of metabolic and respiratory networks.^1−4^ Among multiple Fe–S stoichiometries and coordination, the cubane-type [4Fe-4S] cluster is associated with the lowest reported standard reduction potential down to −645 mV, depending on pH.^5,6^ [4Fe-4S] proteins play essential roles in central carbon metabolism, N_2_ fixation, H_2_ respiration, organohalide respiration, the biogeochemical sulfur cycle, and C_1_ metabolism.^7−12^ Biogenesis of [4Fe-4S] clusters is a complex process involving multiple maturases with long evolutionary histories.^13^ In bacteria, the assembly process follows a scheme of (i) the extraction of the sulfide group from the precursor cysteine; (ii) the assembly of the [4Fe-4S] cluster on a scaffold; and (iii) the transfer of the [4Fe-4S] cluster to recipient proteins.^14^ This scheme can be accomplished by three types of helper protein systems: NIF (nitrogen fixation), ISC (iron-sulfur cluster), and SUF (mobilization of sulfur). The NIF system is responsible for the cubane-type Fe–S cluster assembly of nitrogenase for N_2_ fixation,^15^ while the ISC system is the primary system for Fe–S cluster assembly in bacteria.^16^ The third Fe–S cluster assembly system, the SUF system, is more O_2_-tolerant and is activated when cells suffer from iron starvation and oxidative stress.^17−19^ The SUF system is encoded by the *suf* operon (*sufABCDSE*). The encoded SUF subunits are assembled into two complexes: SufSE and SufBC_2_D. The SufS-SufE pair extracts S^2–^ from cysteine; the SufBC_2_D complex acts as a scaffold, receiving the S^2–^ group from the SufES complex and incorporating Fe^2+/3+^ for Fe–S cluster assembly, which is an ATP- and FADH_2_-consuming process;^20^ the SufA is a Fe–S cluster carrier protein that transfers the Fe–S clusters to the recipient proteins (Figure 1).

Mature [4Fe-4S] proteins have been purified from cells across the tree of life.^21,22^ However, due to the O_2_ lability of [4Fe-4S] clusters, the expression and purification of mature [4Fe-4S] proteins often needs to be performed under strictly anaerobic and highly reduced conditions.^23^ Moreover, purified [4Fe-4S] proteins are often contaminated by ferredoxins (Fd) due to the high affinity of submicromolar *K*_D_ concentrations.^24^ An alternative method for [4Fe-4S] protein maturation is to reconstitute the [4Fe-4S] clusters chemically. For example, the Clostridial apo-Fd was reconstituted into holo-form by incubating apo-Fd with 2-mercaptoethanol, S^2–^, and Fe^2+/3+^ under anaerobic and alkaline conditions.^25^ The traditional chemical reconstitution method has been applied to reconstitute many Fe–S proteins *in vitro* and has reached a level of refinement.^26,27^

Cell-free protein synthesis systems have emerged as a powerful tool for high-throughput protein expression.^28−31^ These cell-free systems are flexible, enabling the adjustment of the environmental conditions to produce correctly folded proteins with cofactors,^32^ and allow the functional expression of enzymes, membrane structural proteins, toxic proteins, and *de novo*-designed polypeptides with high homogeneity. Furthermore, cell-free systems enable protein expression from linear DNA, bypassing the procedures of plasmid cloning and microbe culturing that often cause sequence mutations. Cell lysate-based cell-free systems are easily prepared, robust, and economical, suitable for large-scale, high-yield protein synthesis and high-throughput mRNA translation,^31^ while the reconstituted cell-free systems (i.e., the PURE systems) allow protein synthesis in an environment free of proteases, nucleases, and any undefined contaminated components, suitable for biochemical characterization of complex metabolic networks and the bottom-up reconstruction of synthetic cells.^28,33,34^

Previously, the Swartz group established the protocols for synthesizing mature [2Fe-2S] Fd in a cell extract-based cell-free system (S30) with a high yield of 0.45 mg per mL and a maturation efficacy of ∼85%.^35^ Interestingly, coexpression of the ISC helper proteins in the cells used for cell-extract preparation did not facilitate mature [2Fe-2S] Fd assembly, likely due to the limited ISC expression and activity in host cells under aerobic conditions. Cell-free synthesis of [FeFe] hydrogenase that contains the cubane-type [4Fe-4S] cluster was also attempted by the same group; nevertheless, the maturation efficacy of holo-[FeFe] hydrogenase (44%) was much lower than the [2Fe-2S] Fd even in the presence of the HydEFG helper proteins.^36^ Due to the O_2_ lability of [4Fe-4S] clusters, the cell-free hydrogenase expression and purification were performed under anaerobic conditions, along with the addition of strong reductants to prevent loss of function upon O_2_ exposure.

## Results and Discussion

In this study, we investigated the expression of mature [4Fe-4S] proteins using the PURE system (PUREfrex) and the SUF helper protein system. Ideally, the synthesis of [4Fe-4S] proteins would be possible under aerobic conditions, alleviating the need for complex anaerobic systems. The PUREfrex already includes an efficient ATP regeneration system which can also be used to drive SUF-mediated [4Fe-4S] cluster assembly.^37^ To supply FADH_2_ to the SUF system and to overcome the O_2_ lability of [4Fe-4S] clusters, we designed an O_2_-scavenging cascade including formate dehydrogenase (FDH), NADH-dependent flavin reductase (FRE), and catalase (CAT) to (i) sequentially regenerate FADH_2_ from formate via NADH and (ii) scavenge the dissolved O_2_.

### Stepwise *in Vitro* Reconstitution of the SUF System from Individually Purified SUF Subunits

**Figure 2:**
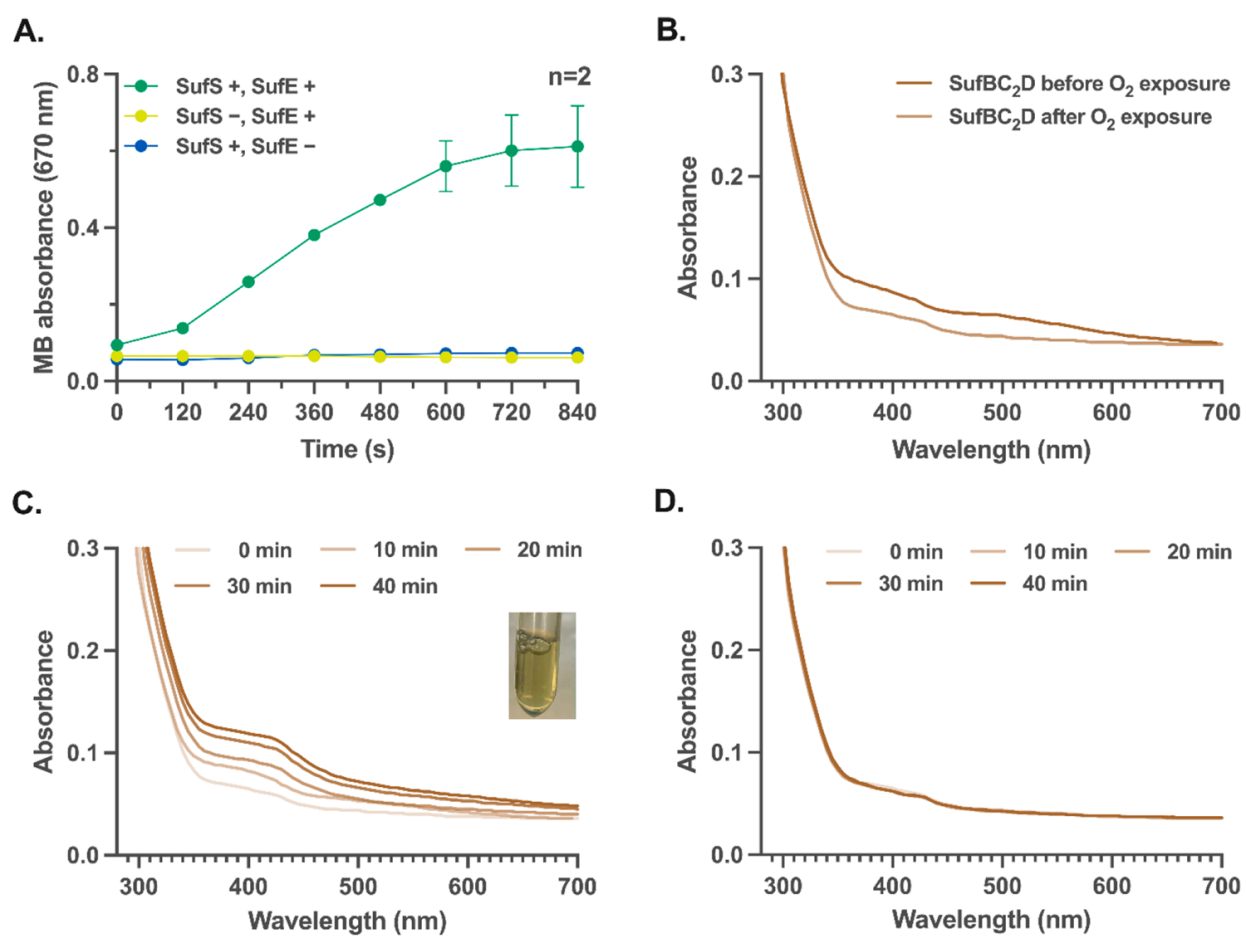
Anaerobic [4Fe-4S] cluster reconstitution using the SUF system. (A) Cysteine desulfurase activity of the SufES complex was measured with and without SufS or SufE. Free S^2–^ production was measured based on the time-dependent production of methylene blue (λ_670 nm_). The bars represent the range and the data points represent the average from two independent experimental replicates, respectively. (B) Observation of [4Fe-4S] clusters (λ_420 nm_) degradation on the reconstituted SufBC_2_D complex after a 10 min aeration. (C,D) SUF-mediated [4Fe-4S] assembly was conducted with the oxidized SufBC_2_D complex in the (C) presence or (D) absence of SufES under anaerobic conditions.

We first sought to reconstitute the SUF system *in vitro* in a stepwise manner using the recombinant SUF subunits individually purified from *E. coli* (Figure S1). According to the literature, the first step in the SUF-mediated Fe–S cluster assembly is S^2–^ extraction from cysteine by the SufES complex.^38^ Indeed, we observed S^2–^ accumulation in reactions when both cysteine and recombinant SufES were added (Figure 2A). Next, we tried to validate the activities of the recombinant SufBC_2_D complex *in vitro*, because previous works generally coexpressed the *sufBCD* and directly purified the mature SufBC_2_D complex from the host via multistep liquid chromatography.^39^ [4Fe-4S] cluster assembly on the SufBC_2_D complex can be inferred from the increase in the characteristic optical absorbance of [4Fe-4S] clusters at 420 nm.^40^ We first reconstituted the SufBC_2_D complex under anaerobic conditions. Notably, a small initial amount of [4Fe-4S] cluster was observed (Figure 2B) and the λ_420 nm_ of the reconstituted SufBC_2_D complex decreased after aeration. We then incubated the oxidized SufBC_2_D complex with cysteine, Fe^2+/3+^, ATP, FADH_2_ (reduced by dithionite), and the recombinant SufES under anaerobic conditions and observed a time-course increase in the λ_420 nm_ (Figure 2C). Moreover, the increase in λ_420 nm_ was not observed in the assays devoid of the recombinant SufES (Figure 2D), suggesting that the recombinant SufBC_2_D complex, along with SufES, is capable of assembling [4Fe-4S] clusters *in vitro* under anaerobic conditions. It is worthwhile mentioning that adding a low Fe^2+/3+^ concentration (<0.1 mM) in the assays prevents the formation of blackish FeS,^41^ which is required for optical measurement in the absence of turbidity (Figure S2).

Next, we validated whether the recombinant SufA is capable of transferring [4Fe-4S] clusters from the SufBC_2_D complex to the recipient proteins *in vitro*. A thermotolerant [4Fe-4S] Fd from the hyperthermophilic archaeon *Thermococcus profundus* was used as [4Fe-4S] cluster recipient protein.^42^ Cytochrome C reduction by Fd was followed at λ_550 nm_, with electrons supplied by a ferredoxin-NADP^+^ reductase system for Fd reduction (Figure S3A).^35^ Often purified [4Fe-4S] Fd results in contaminated Fd-binding proteins, whereas the thermotolerance of *Thermococcus* Fd allows a single-step purification by heating at 92 °C for 10 min (Figure S1). Accordingly, we heterologously expressed and purified holo-Fd from anaerobically cultivated *E. coli* cultures. Following a chemical reconstitution to fully mature the recombinant Fd (Figure S4A), the brown-colored holo-Fd preparations with a defined gradient of concentrations were used to establish the optical cytochrome C reduction assay (Figure S3BC).

Subsequently, we synthesized and purified the recombinant apo-Fd using the PUREfrex free of the SUF system and Fe^2+/3+^ under aerobic conditions. The recombinant apo-Fd was incubated with the recombinant SufBC_2_D complex charged with [4Fe-4S] clusters (Figure 2C) with and without Fe^2+^, cysteine, or the recombinant apo-Fd (Figure S3C). The background activities observed in the negative controls could result from either (i) the trace holo-Fd contamination in the commercial spinach ferredoxin-NADP^+^ reductase (*K*_m_ of Fd < 3 μM) used for Fd reduction in the cytochrome C reduction assays;^43^ and (ii) the chemical reduction by reductants (e.g., Na_2_S and DTT). These data suggested that the reconstituted SUF system (SufABCDSE) can likely mature apo-[4Fe-4S] proteins *in vitro*.

**Figure 3:**
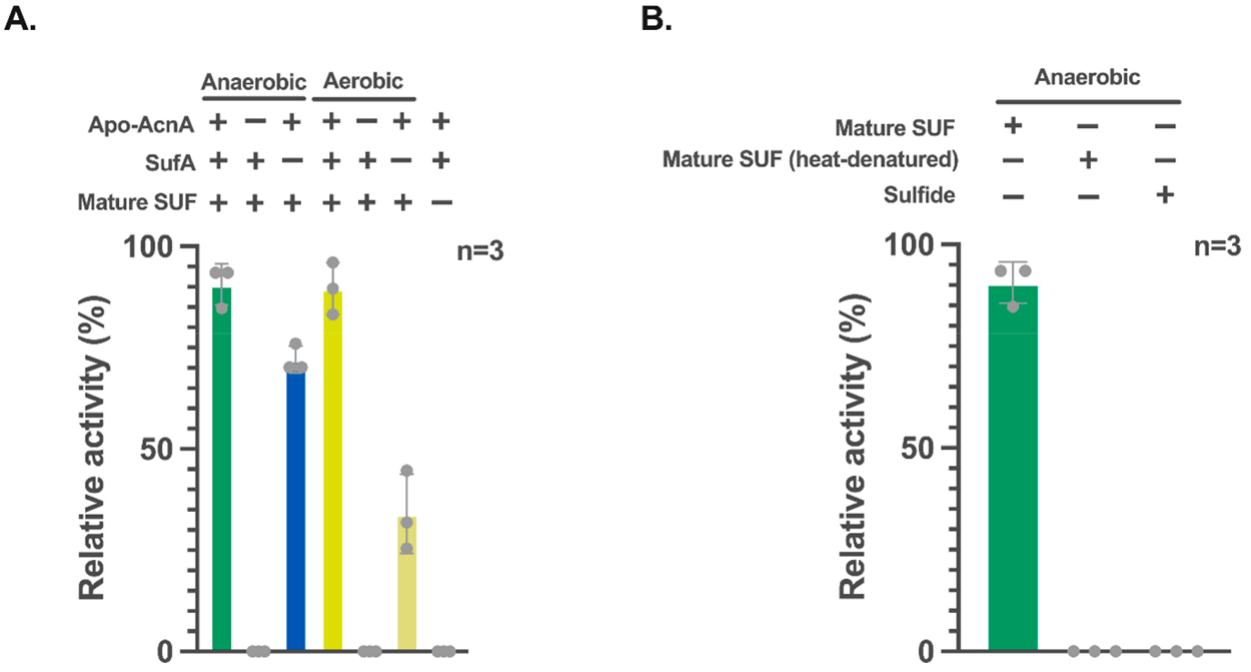
[4Fe-4S] cluster transfer from mature SufBC_2_D complex to apo-AcnA. (A) Apo-AcnA maturation by the SUF system reconstructed under anaerobic and aerobic conditions created by enzymatic O_2_-scavenging system (see later section). (B) Anaerobic holo-AcnA synthesis using mature SUF, heat-denatured SUF system, or S^2–^. The reconstitution efficiency of holo-AcnA is evaluated as a function of the relative enzymatic activity to that of the fully chemically reconstituted AcnA using an isocitrate dehydrogenase activity assay (see Supporting Methods). The bars represent the standard deviations. The dots and columns represent the individual values and mean from three independent experimental replicates, respectively.

To gain a more definite answer, we also used PUREfrex to produce the apo-form of *E. coli* aconitase A (AcnA). The maturation of the SUF system can be validated by an NADP-dependent optical assay (λ_340 nm_) (Figure S5A) where isocitrate, the product of aconitase, is converted to alpha-ketoglutarate by the NADP-dependent isocitrate dehydrogenase, with holo-AcnA purified from anaerobically cultivated *E. coli* cultures as the standard (fully reconstituted via the chemical method) (Figures S4B, S5B,C). Apo-AcnA incubated with the SUF system for 15 min under anaerobic conditions demonstrated an enzyme activity of ∼90% of holo-AcnA activity. No aconitase activity was observed in the reactions without apo-AcnA or the mature SUF (Figure 3A). Moreover, the apo-AcnA incubated with the anaerobically reconstructed SUF system but without SufA demonstrated reduced enzyme activity (∼70%). Furthermore, no aconitase activity was observed when the apo-AcnA was incubated with either heat-denatured SufBC_2_D complex or S^2–^ for 15 min, suggesting that the [4Fe-4S] cluster of holo-AcnA was transferred from the recombinant SUF system but not assembled abiotically under the reduced conditions (Figure 3B). Together, our data revealed that the recombinant SufBC_2_D complex is capable of assembling and subsequently transferring the [4Fe-4S] clusters to the recipient proteins with limited efficiency *in vitro* under anaerobic conditions, and the efficiency can be enhanced by adding the recombinant SufA.

### A Bifunctional O_2_-Scavenging Enzyme Cascade Enabling Aerobic [4Fe-4S] Cluster Assembly

**Figure 4:**
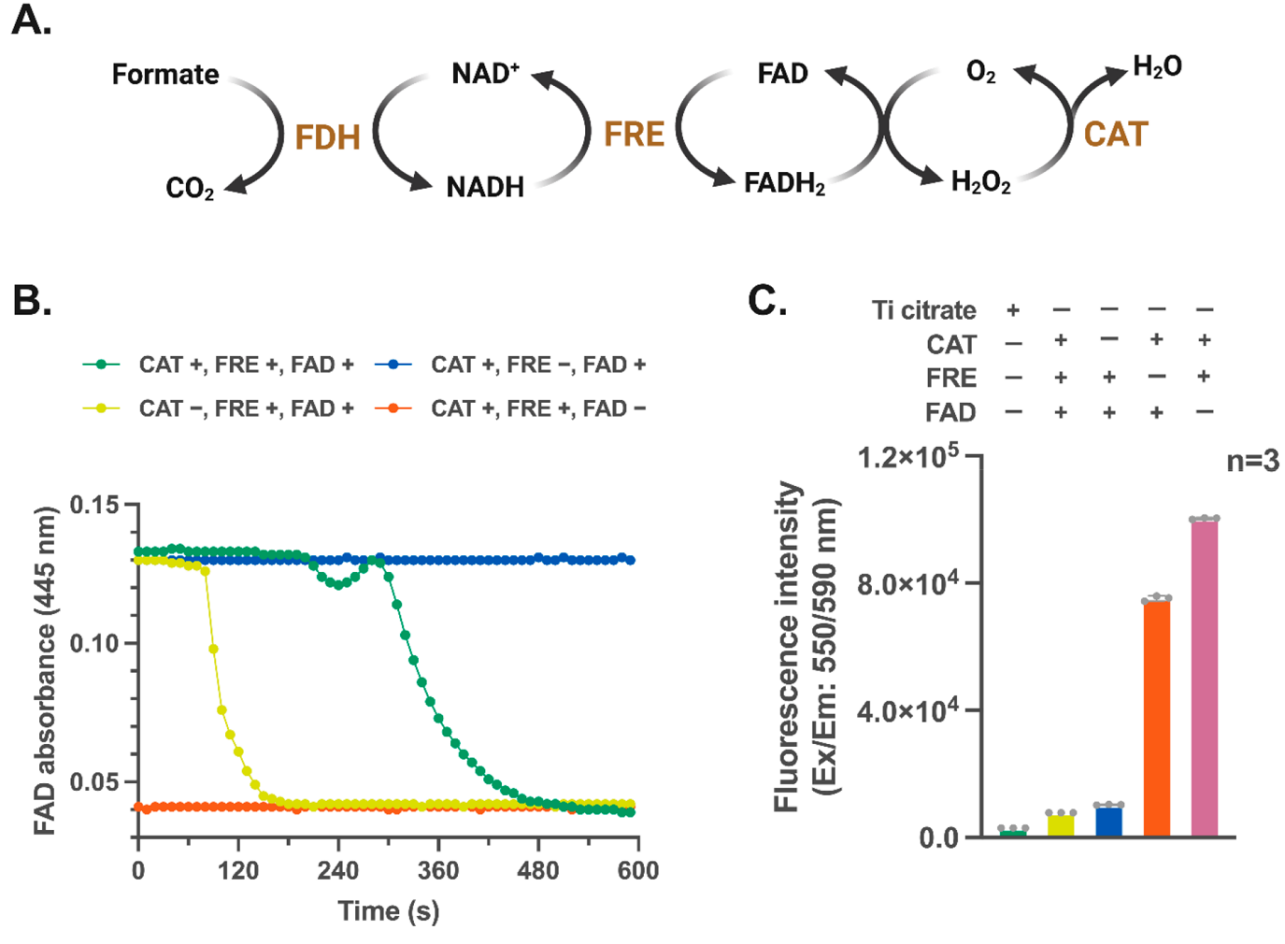
The bifunctional enzyme cascade for FADH_2_ regeneration and O_2_ scavenge. (A) Schematic diagram showing the bifunctional O_2_-scavenging enzyme cascade. (B) FADH_2_ production was monitored by a time-dependent decrease in λ_445 nm_ (0–10 min). The reactions were conducted with and without catalase (CAT), flavin reductase (FRE), or FAD. (C) The anaerobicity of the reaction mixture was evaluated by an oxidation–reduction indicator, resorufin. The bars represent the standard deviation. The dots and columns represent the individual value and mean from three independent experimental replicates, respectively.

Following *in vitro* reconstitution of the mature SUF system under anaerobic conditions, we then worked to overcome the O_2_ lability of [4Fe-4S] clusters by amendment with an O_2_-scavenging system. Inherent from the O_2_-lability of [Fe–S] proteins, several enzymatic O_2_-scavenging approaches have been developed during the past few decades. Catalase, glucose oxidase, and protocatechuate dioxygenase represent the most common O_2_-scavenging enzymes.^44,45^ However, in our case, their substrates/products (i.e., gluconolactone and protocatechuate, respectively) are UV–vis active, which affects the optical analysis for monitoring the [4Fe-4S] cluster assembly. Recently, bilirubin oxidase has been used as a single-enzyme O_2_-scavenger without H_2_O_2_ generation.^46^ Unfortunately, bilirubin (red) and the oxidized product biliverdin (green) also affect the optical analysis of the [4Fe-4S] clusters. Interestingly, the NADH-dependent flavin reductase (FRE) required for FADH_2_ regeneration in the SUF system can also scavenge O_2_ to form H_2_O_2_.^47^ Working with catalase (CAT), the translucent FADH_2_ can sequentially reduce the level of O_2_ to H_2_O via H_2_O_2_ (O_2_ + 2 FADH_2_ → 2 H_2_O + 2 FAD). Therefore, we employed flavin reductase and catalase for O_2_-scavenging. The NADH required for FADH_2_ regeneration was supplied by the NAD^+^-dependent formate dehydrogenase (FDH) using formate as the donor of reducing equivalent (Figure 4A).^48^ Accordingly, we have purified *E. coli* FRE/CAT and *Pseudomonas* FDH (Figure S1) to test if this bifunctional three-enzyme cascade can function in the buffer system of the PUREfrex. We measured time-course NAD^+^ reduction (λ_340 nm_) by adding both formate and formate dehydrogenase to the PUREfrex buffer (Figure S6AB). The addition of both flavin reductase and FAD to the PUREfrex buffer resulted in a time-course FAD reduction (λ_445 nm_) (Figures 4B and S6C), and the yellow color of the PUREfrex buffer gradually faded away to become translucent. The same reaction mixture with catalase addition demonstrated a delayed FAD reduction compared to the catalase-free mixture, suggesting FADH_2_ was recycled back to FAD. Likely, the produced FADH_2_ was mainly consumed for O_2_ degradation in the catalase-added PUREfrex buffer in the first 8 min. Together, these data suggest that the O_2_-scavenging enzyme cascade can sustain continuous FADH_2_ regeneration in the PUREfrex buffer system under aerobic conditions.

**Figure 5:**
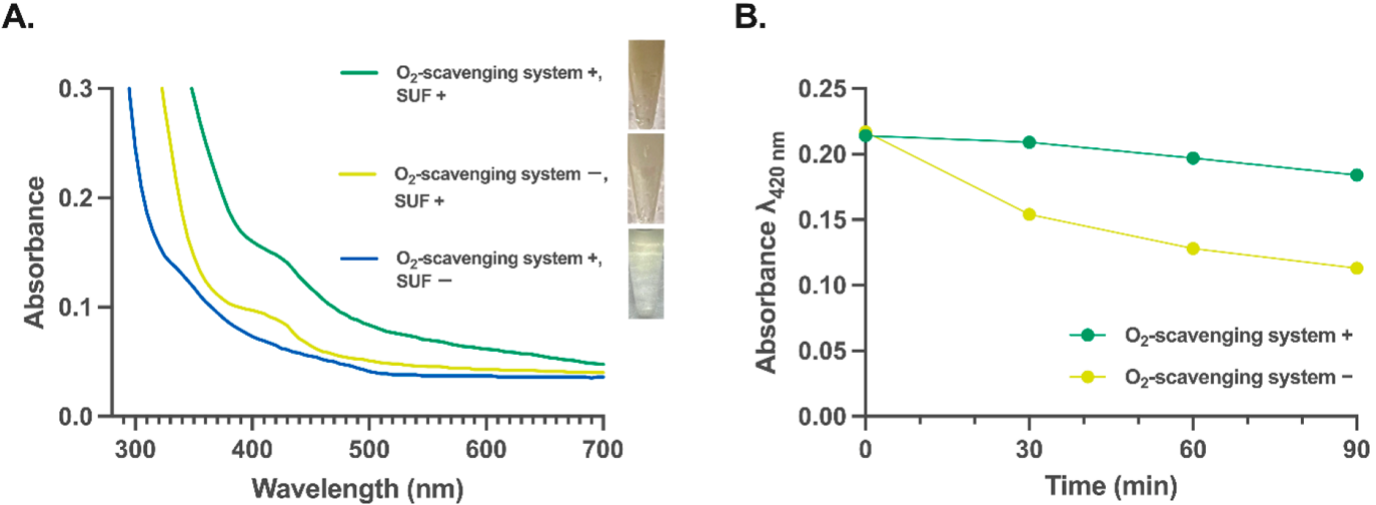
The bifunctional O_2_-scavenging enzyme cascade enables SUF-mediated [4Fe-4S] cluster assembly under aerobic conditions. (A) The SUF-mediated [4Fe-4S] assembly (based on the increase in λ_420 nm_) with and without the O_2_-scavenging system under aerobic conditions. (B) The bifunctional O_2_-scavenging enzyme cascade protects the [4Fe-4S] cluster from oxidation (based on the decrease in λ_420 nm_) under aerobic conditions. The degradation and formation of [4Fe-4S] clusters were monitored by the UV–vis spectrophotometric analysis (280–700 nm).

Next, we employed resorufin, a sensitive fluorescent O_2_ indicator, to assess the O_2_-scavenging capability of the O_2_-scavenging cascade. Under reduced conditions (i.e., dissolved O_2_ removed), the pink-colored and highly fluorescent resorufin would undergo a reversible reduction to form the nonfluorescent dihydroresorufin (standard reduction potential ∼ −51 mV).^49^ Consistent with the consumption of O_2_, the PUREfrex buffer with the O_2_-scavenging enzyme cascade demonstrated a fluorescence emission (λ_590 nm_) much lower than reactions devoid of either flavin reductase or FAD (Figure 4C). Moreover, the fluorescence emission of the PUREfrex buffer amended with the O_2_-scavenging enzyme cascade is comparable to that of the titanium citrate-reduced reactions. These data suggested a faster rate of O_2_ scavenging by the enzyme cascade than the rate of diffusion of the O_2_ into the PUREfrex buffer. Therefore, we amended this O_2_-scavenging enzyme cascade to the SUF-incorporated PUREfrex buffer and performed the SUF-mediated [4Fe-4S] assembly *in vitro* under aerobic conditions. After 30 min of incubation, the color of SUF-incorporated PUREfrex buffer turned brown, along with an obvious increase in the characteristic optical absorbance of [4Fe-4S] clusters at λ_420 nm_, comparable to that of the SUF-incorporated PUREfrex buffer incubated anaerobically (Figure 5A). Furthermore, the O_2_-scavenging enzyme cascade also protected the [4Fe-4S] cluster from oxidation under aerobic conditions for at least 1.5 h, while the absence of the enzyme cascade resulted in a rapid oxidation of the [4Fe-4S] cluster in the first 30 min (Figure 5B).

The combination of the O_2_-scavenging enzyme cascade and the SUF system enabled *in vitro* [4Fe-4S] cluster reconstitution under aerobic conditions. We have also shown that mature SUF reconstituted using O_2_-scavanging system can also support Apo-AcnA maturation (Figure 3A). In *E. coli*, flavin reductase FRE is the main enzyme responsible for FMNH_2_/FADH_2_ regeneration, i.e., the native enzyme supporting the [4Fe-4S] cluster assembly by the SufBC_2_D complex.^20,47^ Moreover, our O_2_-scavenging system only employs a low FAD concentration (∼20 μM) comparable to the intracellular FMN/FAD concentration in *E. coli*.^50^ One can be envisaged that such a biomimetic system for [4Fe-4S] protein maturation would be suitable and compatible to reconstruct the cell-wide metabolic networks in both cell extract-based and reconstituted cell-free systems. Going further, the required O_2_-scavenging capacity, which is proportional to the formate concentration, can be estimated based on the ideal gas law (*pv* = *nRT*), atmospheric O_2_ content (20%), and Henry’s law constant of O_2_ (H_aq/gas_ = 0.032). For example, since our PUREfrex reactions were performed in a capped 0.3 mL reaction tube with 0.1 mL of the headspace of the atmosphere, the amount of O_2_ in the PUREfrex reactions would be ∼0.9 μmol/tube (see the detailed calculation in Table S1). Given that the O_2_-scavenging enzyme cascade reduces each O_2_ molecule at the cost of two FADH_2_, complete O_2_ removal would require ∼1.8 μmol (9 mM) of FADH_2_ (i.e., formate). Excessive formate addition to the system, while securing the anaerobicity, would result in a drastic pH shift.

### One-Pot, Two-Step Cell-Free Synthesis of Active Aconitase under Aerobic Conditions

**Figure 6:**
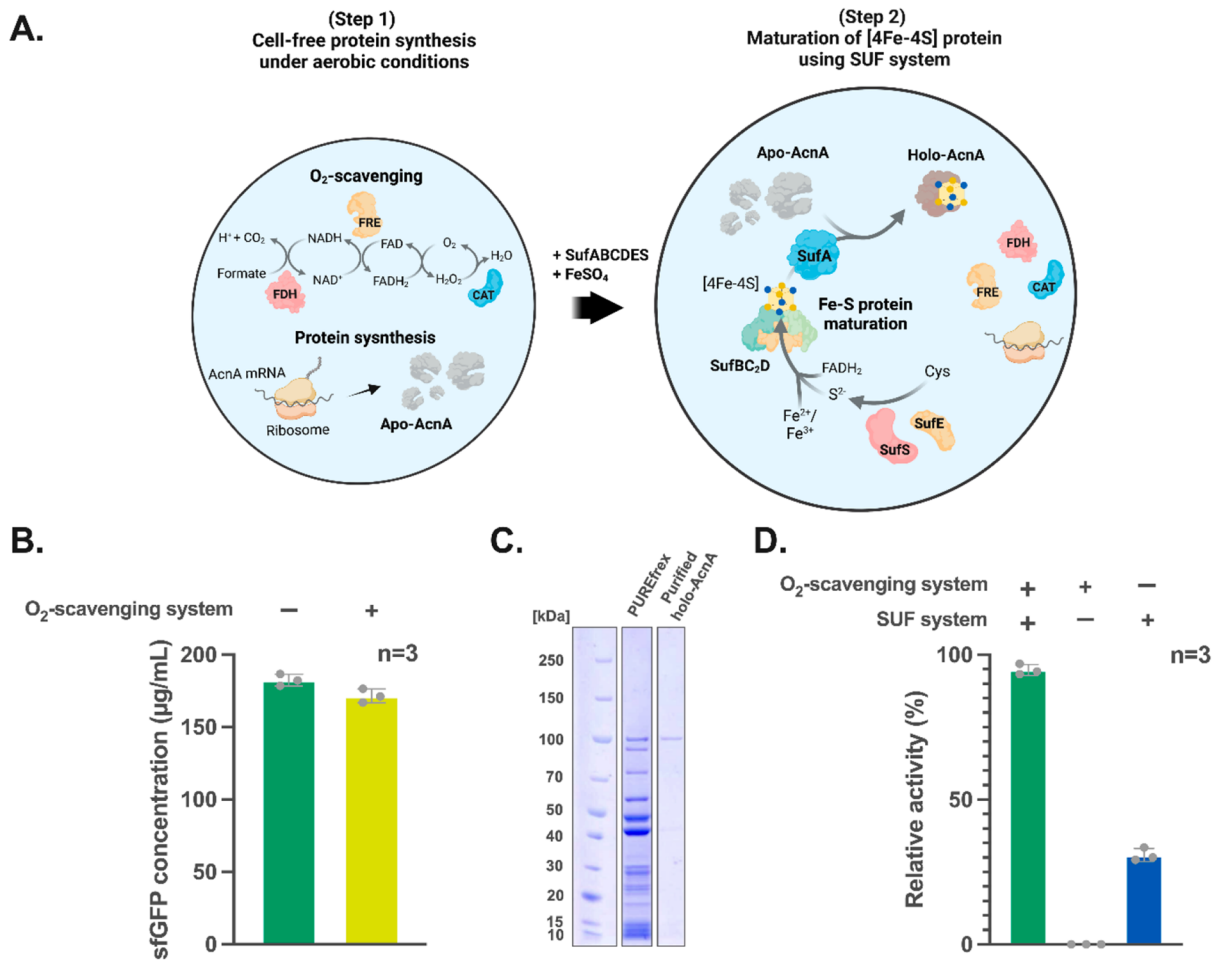
One-pot *de novo* synthesis of mature [4Fe-4S] protein under aerobic conditions. (A) Schematic diagram showing one-pot two-step cell-free synthesis of mature [4Fe-4S] cluster bearing AcnA under aerobic conditions. *In vitro* translation of AcnA mRNA is carried out in a test tube along with the three O_2_-scavanging enzymes (Step 1). FeSO_4_ and SUF components are further added to drive maturation of the apo-AcnA to its holo-form (Step 2). Other required components such as cysteine (Cys) and ATP are carried over from the PUREfrex reaction. (B) Superfolder GFP (sfGFP) production in the PUREfrex reaction mixtures with and without the O_2_-scavenging enzyme cascade after 6 h of incubation. (C) Purification of holo-AcnA free of N-His_6_-tag verified by SDS-PAGE analysis. (D) Relative activity of AcnA in the one-pot PURE system with and without the SUF and O_2_-scavanging system. The bars represent the standard deviations. The dots and columns represent the individual values and mean from three independent experimental replicates, respectively.

Finally, we tested the possibility of combining the processes of mRNA translation, O_2_ scavenging, and [4Fe-4S] cluster maturation into a one-pot process. Due to the adverse effects of S^2–^ (metal precipitation) and Fe^2+^ (in-line cleavage of RNA^51^ and generation of reactive oxygen species) during cell-free translation, we separated the *in vitro* translation and cluster reconstruction process into two steps (Figure 6A). In step 1, AcnA mRNA was added to the PUREfrex along with the three enzymes for O_2_-scavenging (FDH/FRE/CAT) to maintain anoxic condition while producing apo-AcnA. In step 2, FeSO_4_ and SufABCDSE were added to the reactions for the maturation of the apo-AcnA. This two-step process can prevent continuous cysteine desulfurization by SufES that would result in (i) a decrease of available cysteine for mRNA translation and (ii) metal (e.g., Fe^2+^) precipitation by the S^2–^.

For a successful one-pot synthesis, a prerequisite is the compatibility of PUREfrex with the O_2_-scavenging enzyme cascade. The incompatibility of the two components can lead to a significant reduction of the protein yield. Therefore, we first used the mRNA of superfolder green fluorescent protein (sfGFP) as an indicator of the protein yield in PUREfrex and compared the sfGFP yield of the PUREfrex reactions with and without the O_2_-scavenging enzyme cascade, respectively. Our data showed that the incorporation of the O_2_-scavenging enzyme cascade to the PUREfrex only resulted in a reduction of sfGFP yield <5% (Figure 6B), suggesting that the two components are compatible. Another prerequisite is the maturation of the SUF system to provide [4Fe-4S]-charged SufBC2D complex in the O_2_-scavenged PUREfrex buffer under aerobic conditions (Figure 5A). Accordingly, we translated apo-AcnA mRNA using PUREfrex along with the O_2_-scavenging system (Figure 6A, Step 1), followed by an addition of SUF components and FeSO_4_ to generate holo-AcnA (Figure 6B, Step 2). Use of an HRV protease for N-His_6_-tag removal allowed us to further purify holo-AcnA from other recombinant proteins in the PUREfrex reaction mixture (Figure 6C). The purified holo-AcnA demonstrated a relative enzyme activity of ∼95% to that of the fully chemically matured holo-AcnA (Figure 6D). Moreover, liquid chromatography–mass spectroscopy (LC-MS) analysis revealed concurring citrate consumption and alpha-ketoglutarate production in the assays with AcnA purified from the one-pot, two-step system but not in the assays with AcnA purified from the PUREfrex reactions devoid of the SUF system (Figure S7 and S8). Interestingly, the AcnA produced from the SUF-amended PUREfrex reactions without the O_2_-scavenging enzyme cascade demonstrated a relative enzyme activity of ∼30% of the aconitase activity (Figure 6D), suggesting that consistent with the observation of previous studies, the SUF system is relatively O_2_-tolerant. Altogether, these data suggested the successful production of holo-aconitase from the one-pot, two-step process.

## Conclusions

Functional expression and purification of [4Fe-4S] proteins have been long-term challenges in biochemical research. In this study, we demonstrated *in vitro* assembly and transfer of [4Fe-4S] clusters from Fe^2+/3+^ and S^2–^ from cysteine using the SUF system reconstituted from individually purified SUF subunits. The O_2_ tolerance of the SUF helper proteins allows facile heterologous expression and purification of each subunit under aerobic conditions and later reconstitution. Interestingly, based on the results of the *Thermococcus* Fd-mediated cytochrome C reduction assays, the *E. coli* SUF system seems to be capable of transferring the [4Fe-4S] clusters to [4Fe-4S] proteins from other organisms, suggesting broad applicability of the reconstituted SUF system. In conclusion, the PUREfrex supplied with both the SUF system and the bifunctional O_2_-scavenging enzyme cascade serves as a facile and efficient platform for synthesizing O_2_-labile [4Fe-4S] proteins. We anticipate that this renovated PURE system, with the flexibility to be paired with other helper proteins, will facilitate future studies on more challenging [4Fe-4S] proteins, such as FeMoco-dependent nitrogenase, cobalamin-dependent dehalogenases, and NiFeS acetyl-CoA synthase/CO dehydrogenase. Furthermore, although not fully demonstrated in this study, the amendment of the O_2_-scavenging enzyme cascade also makes the PURE system redox-active. If paired with ferredoxin-NADP^+^ reductase, the four-enzyme cascade can support the regeneration of all common redox equivalents in cells (Fd, NADPH, cytochrome C, ubiquinone, and FMNH_2_/FADH_2_). Therefore, we also foresee the compatibility of this renovated PURE system with the *in vitro* reconstitution of complex biosynthesis pathways and respiratory networks, likely to the extent of *de novo* artificial cell reconstruction.

## Methods

Methods for heterologous protein expression and purification, *in vitro* RNA transcription, the optical enzyme activity assays, and analytical chemical methods are shown in the Supporting Information. All experiments requiring the anaerobic environment were performed in a Coy Lab type B vinyl anaerobic chamber with an atmosphere of a 5:95% (v/v) H_2_:N_2_ gas mixture. *K*_m_ and *k*_cat_ values of the enzymes used in this work are given in Table 1.

**Table 1:** *K*_m_ and *k*_cat_ of the Enzymes Used in This Work^a^

enzyme name	substrate	*K*_m_ (mM)	*k*_cat_ (s)	specific activity (μmol/min/mg)
Isocitrate dehydrogenase	Isocitrate	0.0075	58	38
	NADP^+^	0.0055	32	
Ferredoxin-NADP^+^ reductase	NADPH	0.003	250	–
	Fd_(ox)_	0.05	0.15	
Cysteine desulfurase (SufS)	l-Cysteine	0.05	0.2	0.01
Aconitase (AcnA)	Citrate	1.15	–	6.0
Flavin reductase (FRE)	FAD	0.001	40	–
	NADH	0.18	40	
Formate dehydrogenase (FDH)	Formate	7.5	10	–
	NAD^+^	0.05	7.3	

a The values were referred from the BRENDA database.^55^

### Experimental Materials

All chemicals were purchased from Sigma-Aldrich (St. Louis, MO, USA) unless specified otherwise. The PUREfrex reagents were provided by GeneFrontier Corp. (Kashiwa, Chiba, Japan). The pUC57 plasmids carrying the recombinant *E. coli* SUF proteins, *Thermococcus* Fd, and the pET-21d(+) plasmid carrying the recombinant *E. coli* catalase (CAT) were purchased from GenScript Biotech Corporation (Nanjing, Jiangsu, China). The pET-23b(+) plasmid carrying the recombinant *Pseudomonas* formate dehydrogenase gene is a gift from Prof. Alexander F. Yakunin, Bangor University, United Kingdom.^48^ The ASKA *E. coli* clones for aconitase (AcnA) and flavin reductase (FRE) were purchased from the National Institute of Genetics (SHIGEN) (Mishima, Shizuoka, Japan).^52^ The detailed information for the corresponding recombinant proteins is shown in Table S2.

### Gene Cloning

The *suf* genes (*sufA*/*sufB*/*sufC*/*sufD*/*sufE*/*sufS*) were cloned into a pET-26b(+) expression vector by Seamless ligation cloning extract (SLiCE) cloning,^53^ while *acnA* gene was cloned into pET-23a(+) for *in vitro* transcription. The linearized vector was amplified from the pET-26b(+)/pET-23a(+) plasmid DNA using the appropriate forward and reverse primers. The *suf* genes were amplified from pUC57 plasmid DNA, while *acnA* gene was amplified from the corresponding ASKA *E. coli* clone. Note that the HRV protease-cutting site was inserted between the genes of N-His_6_-tag and *acnA* by PCR. All primer sequences are available in Table S2. The SLiCE cloning reaction was performed as described previously.^54^ Briefly, SLiCE solution (1 μL) and 10× SLiCE buffer (1 μL) were mixed with 60 ng of the inset DNA and 60 ng of the linearized pET-26b(+) vector (C-His_6_ tag removed by PCR), and the final volume of the SLiCE reaction was adjusted to 10 μL using sterilized ddH_2_O. The reaction mixture was incubated at 37 °C for 15 min to produce the pET-26b(+)-SufA/SufB/SufC/SufD/SufE/SufS and pET-23a(+)-AcnA plasmids, respectively. The pET-26b(+)-SufA/SufB/SufC/SufD/SufE/SufS plasmids were transformed into *E. coli* BL21(DE3) cells (New England Biolabs, Inc., Ipswich, MA, USA) for heterologous protein overexpression, respectively, while the pET-23a(+)-AcnA plasmid was subsequently amplified by PCR to make a linear DNA harboring the T7 promoter for the following *in vitro* transcription.

### *In Vitro* Assembly of the [4Fe-4S] Cluster on the SufBC_2_D Complex

#### i Anaerobic Conditions

The [4Fe-4S]-charged SufBC_2_D complex was anaerobically produced inside the anoxic chamber. Reaction mixture containing HEPES-Na^+^ (pH 7.6; 50 mM), NaCl (100 mM), MgCl_2_ (5 mM), dithiothreitol (DTT) (1 mM), pyridoxal 5′-phosphate (10 μM), ATP (1 mM), SufE (0.5 μM), SufS (0.5 μM), SufB (10 μM), SufC (20 μM), SufD (10 μM), FeSO_4_ (0.1 mM), cysteine (0.5 mM), and glycerol (5%, v/v). The reaction was initiated by the addition of cysteine and incubated at 37 °C for 1 h. Optical analysis of the SufBC_2_D complex was performed using a microplate reader (Epoch 2 microplate reader, Agilent, CA, USA). The mature SUF including [4Fe-4S]-charged SufBC_2_D complex was further purified using Ni-sepharose-based immobilized affinity chromatography, as described in Supporting Methods. The protein stock solutions were flash-frozen with liquid N_2_ and stored at −80 °C.

#### ii Aerobic Conditions

The [4Fe-4S]-charged SufBC_2_D complex was aerobically produced on the benchtop using O_2_-scavanging system. Reaction mixture containing HEPES-Na^+^ (pH 7.6; 50 mM), NaCl (100 mM), MgCl_2_ (5 mM), DTT (1 mM), pyridoxal 5′-phosphate (10 μM), ATP (1 mM), sodium formate (10 mM), NADH (0.1 mM), FAD (20 μM), formate dehydrogenase (0.05 mg/mL), flavin reductase (0.05 mg/mL), catalase (0.01 mg/mL), SufE (0.5 μM), SufS (0.5 μM), SufB (10 μM), SufC (20 μM), SufD (10 μM), FeSO_4_ (0.1 mM), cysteine (0.1 mM), and glycerol (5%, v/v). The reaction mixture amended with the O_2_-scavenging enzyme cascade was first assembled and incubated at 37 °C for 10 min to scavenge the dissolved O_2_. The SUF proteins were then added to the reaction mixture. The reaction mixture was incubated at 37 °C for 60 min. Optical analysis of the [4Fe-4S]-charged SufBC_2_D complex was performed by using a microplate reader (Epoch 2 microplate reader, Agilent, CA, USA). The mature SUF including [4Fe-4S]-charged SufBC_2_D complex was further purified using Ni-sepharose-based immobilized affinity chromatography as described in Supporting Methods. The reaction mixture was flash-frozen in liquid N_2_ and stored at −80 °C for the following *in vitro* maturation of [4Fe-4S] proteins.

### *In Vitro* Maturation of Apo-Ferredoxin and Apo-Aconitase Using the SUF System under Anaerobic Conditions

Apo-ferredoxin (Fd) and apo-aconitase (AcnA) were synthesized by PUREfrex2.0 as described in Supporting Methods. The apoproteins were then mixed with purified mature SUF inside the anoxic chamber. The reaction mixture contained HEPES-K^+^ (pH 7.6; 50 mM), DTT (0.5 mM), 2-mercaptoethanol (0.1%, v/v), FeCl_3_ (40 μM), anaerobically/aerobically produced SufBC_2_D complex (2 μM), SufA (1.5 μM), apo-Fd/AcnA (0.01–0.1 mg/mL), and glycerol (5%, v/v). Note that for control experiments, heat-denatured SUF was prepared by incubating the mature SufBC_2_D complex and SufA at 95 °C for 10 min and was added to the reaction as well as S^2–^ (8 μM) was added to the reaction instead of the SUF system. The reaction mixture was then incubated at 37 °C for 10 min and dialyzed using the Amicon Ultra-0.5 centrifugal filter (cutoff: 3 kDa) (Merck KGaA, Darmstadt, Germany) inside the anaerobic chamber.

### The Bifunctional Enzyme Cascade for FADH_2_ Regeneration and O_2_ Scavenging

The O_2_-scavenging enzyme cascade reaction was performed in the reaction mixture (100 μL) with HEPES-Na^+^ (pH 7.6; 50 mM), NaCl (100 mM), MgCl_2_ (5 mM), sodium formate (10 mM), NADH (0.1 mM), FAD (20 μM), formate dehydrogenase (0.05 mg/mL), flavin reductase (0.05 mg/mL), catalase (0.01 mg/mL), and glycerol (5%, v/v). The reaction was initiated by the addition of formate dehydrogenase. Assays were performed under aerobic conditions using an optical 96-well microplate at 37 °C for 10 min with a UV–vis scanning at λ_445 nm_ every 5 s on a microplate reader. The stock solution of sodium formate (1 M) was adjusted to pH 7.6 in a HEPES-Na^+^ buffer (50 mM) to prevent a drastic pH shift in the reaction mixture.

### One-Pot Cell-Free Synthesis of Holo-Aconitase under Aerobic Conditions

Apo-AcnA was synthesized using a customized PUREfrex2.0 system following the manufacturer’s standard protocols on a benchtop. Briefly, in an ice bath, solution I (amino acids, NTPs, tRNAs and substrates for enzymes, etc.; 100 μL), solution II (enzyme mixtures; 10 μL), and solution III (ribosome; 20 μL) were mixed with the components to final concentrations of flavin reductase (0.05 mg/mL), formate dehydrogenase (0.05 mg/mL), catalase (0.025 mg/mL), sodium formate (10 mM), pyridoxal 5′-phosphate (10 μM), NADH (0.2 mM), FAD (20 μM), and AcnA mRNA (0.4–0.7 μM) to a final volume of 200 μL. Cell-free protein synthesis was performed at 37 °C for 6 h, and the reaction mixtures were kept on ice after incubation for *in vitro* maturation of aconitase using the SUF system. The PUREfrex reaction mixture containing apo-AcnA was supplemented with FeSO_4_ (0.1 mM) and individually purified SUF subunits: SufA (1.5 μM), SufB (8 μM), SufC (16 μM), SufD (8 μM), SufE (0.4 μM), and SufS (0.4 μM), and was incubated at 37 °C for 10 min. The holo-AcnA was purified using Ni-sepharose-based immobilized affinity chromatography as described in Supporting Methods.

## Supporting Information Available

Table S1. Calculation of dissolved oxygen in the PUREfrex reactions (XLSX)
Supporting Methods; Table S2. Complete list of recombinant proteins, expression vectors, and primers for gene cloning used in this work; Figure S1. SDS-PAGE gel images of purified recombinant proteins and apo-Fd/AcnA synthesized by the PURE system; Figure S2. Images of the mature SufBC_2_D complex at different concentrations of FeSO_4_; Figure S3. Standard curve to estimate the holo-Fd concentration using the cytochrome C-coupled optical assays; Figure S4. UV–vis analysis of the recombinant holo-Fd and AcnA purified from the anaerobically cultivated *E. coli* BL21 cells; Figure S5. Standard curve to estimate the holo-AcnA concentration using the aconitase A and isocitrate dehydrogenase-coupled activity assays; Figure S6. NADH and FADH_2_ production using formate dehydrogenase and the O_2_-scavenging system, respectively; Figure S7. LC-MS spectra of citrate; Figure S8. LC-MS spectra of alpha-ketoglutarate (PDF)

## Abbreviations

Fd: ferredoxin
PURE system: reconstituted cell-free protein synthesis system.
