# Supplementary material for: One-Pot De Novo Synthesis of [4Fe-4S] Proteins Using a Recombinant SUF System under Aerobic Conditions
Source: ACS Synth Biol. 2023 Jul 19;12(10):2887–96. doi: 10.1021/acssynbio.3c00155 (PMC10594875; doi:10.1021/acssynbio.3c00155)
Supplement: Supplementary file 2 — sb3c00155_si_002.pdf [file sb3c00155_si_002.pdf]

Supporting Information

One-Pot *De Novo* Synthesis of [4Fe-4S] Proteins Using a Recombinant SUF System under Aerobic Conditions

Po-Hsiang Wang[ab‡*], Shota Nishikawa[cd‡], Shawn Erin McGlynn[ce], and Kosuke Fujishima[cf*]

[a] Department of Chemical Engineering and Materials Engineering, National Central University, Taoyuan 32001, Taiwan.

[b] Graduate Institute of Environmental Engineering, National Central University, Taoyuan 320, Taiwan.

[c] Earth-Life Science Institute, Tokyo Institute of Technology, Tokyo 152-8550, Japan.

[d] School of Life Science and Technology, Tokyo Institute of Technology, Tokyo 152-8550, Japan.

[e] Blue Marble Space Institute of Science, Seattle, Washington 98154, U.S.A.

[f] Graduate School of Media and Governance, Keio University, Fujisawa, 252-0882, Japan

[‡] P.-H.W. and S.N. contributed equally to this work.

[*] To whom correspondence should be addressed. E-mails: pohsiang@ncu.edu.tw; fuji@elsi.jp

Supporting Methods.

Heterologous protein overexpression and purification. Heterologous protein overexpression and purification were performed based on the established protocols published previously with some modifications[1]. Briefly, the cultures were grown under aerobic conditions at 37℃ in a shaker using Terrific Broth (200 rpm) to the optical density of 0.4–0.6 at $OD_{600\ nm}$, followed by the induction using isopropyl β-D-1-thiogalactopyranoside (IPTG) (0.4 mM) and overnight incubation at 16℃. For *Thermococcus profundus* ferredoxin (Fd) and *Escherichia coli* aconitase A (AcnA) expression, after 2 h of the IPTG induction, the cultures were transferred to 1-L screw-capped glass bottles and were supplemented with cysteine (0.2 mM) and $FeCl_3$ (0.1 mM), followed by overnight incubation at 37℃ for Fd and at 16℃ for AcnA, respectively, with constant stirring. Recombinant proteins were purified to at least 95% homogeneity using Ni-sepharose-based immobilized affinity chromatography as previously described[2], and protein purity was analyzed by SDS-PAGE (5–20% SDS-PAGE gels; Supercep™ Ace; Fujifilm Wako Pure Chemical, Osaka, Japan) followed by Coomassie staining (**Figure S1**). Briefly, cells were collected by centrifugation at 8,000 × g for 10 min at 4℃, suspended in the binding buffer: HEPES-Na (pH 7.5; 25 mM), NaCl (0.5 M), glycerol (5%, v/v), and imidazole (5 mM), and the cells were homogenized via sonication in an ice bath for 10 min (1 s on and 2 s off). For Fd, the lysate was subsequently boiled for 20 min at 92℃ to denature the endogenous proteins. The lysate was centrifuged at 20,000 × g for 30 min at 4℃ and the supernatant was passed through a glass gravity column containing approximately 0.5 mL Ni Sepharose™ High Performance resin (GE Healthcare, IL, USA). After washing with the wash buffer: HEPES-Na (pH 7.5; 25 mM), NaCl (0.5 M), glycerol (5%, v/v), and imidazole (25 mM), recombinant proteins were eluted using the elution buffer: HEPES-Na (pH 7.5; 25 mM), NaCl (0.5 M), glycerol (5%, v/v), and imidazole (250 mM). For Fd and holo-AcnA, $FeSO_4$ (0.1 mM) and Na_2S (0.8 mM) were supplemented to the lysate and wash buffer to chemically reconstitute Fe-S cluster on the proteins. The protein stock solutions were flash-frozen in liquid N_2 and stored at −80℃. The N-terminal His_6-tag was retained for all experiments except the experiment for purification of the holo-aconitase A synthesized by the PUREfrex.

Purification of holo-aconitase A. Holo-AcnA was purified from the one-pot cell-free reaction under anaerobic conditions. The one-pot cell-free reaction mixture was passed through a glass gravity column containing approximately 0.1 mL of Ni-sepharose resin. After washing with a wash buffer: HEPES-Na (pH 8.0; 25 mM), NaCl (0.1 M), $FeSO_4$ (40 µM), 2-mercaptoethanol (0.1%, v/v), and glycerol (5%, v/v), HRV 3C Protease (1 U, Takara) was added to the resin to cleave the tag sequence from AcnA and elute the protein. The protein cleavage reaction was performed at 4℃ for 16 h and later the eluate was collected by centrifugation. The eluate was flash-frozen in liquid N_2 and stored at −80℃.

Preparation of apo-ferredoxin and apo-aconitase. Apo-Fd and apo-AcnA devoid of the [4Fe-4S] clusters were synthesized using a PUREfrex2.0 following the manufacturer's standard protocols (https://purefrex.genefrontier.com/products/cellfreeproteinsynthesiskits/purefrex2.0.html). Briefly, in an ice bath, solution I (amino acids, NTPs, tRNAs and substrates for enzymes, etc.; 10 µL), solution II (enzyme mixtures; 1 µL), and solution III (ribosome; 2 µL) were mixed with the mRNAs (0.4–0.7 µM) to a final volume of 20 µL. Cell-free protein synthesis was performed at 37℃ for up to 6 h and the reaction mixture was kept on ice after incubation before further SDS-PAGE analysis or reconstitution experiments.

sfGFP synthesis using a cell-free synthesis system amended with the bifunctional $FADH_2$ regeneration/O_2-scavenging enzyme cascade. Superfolder green fluorescent protein (sfGFP) was synthesized using a customized PUREfrex2.0 system following the manufacturer's standard protocols. Briefly, in an ice bath, solution I (amino acids, NTPs, tRNAs and substrates for enzymes, etc.; 100 µL), solution II (enzyme mixtures; 10 µL), and solution III (ribosome; 20 µL) were mixed with the components to final concentrations of flavin reductase (0.05 mg/mL), formate dehydrogenase (0.05 mg/mL), catalase (0.025 mg/mL), sodium formate (10 mM), pyridoxal 5′-phosphate (10 µM), NADH (0.2 mM), FAD (20 µM), and sfGFP mRNA (0.4–0.7 µM) to a final volume of 200 µL. Note that the reaction mixture was assembled in a 0.2 mL tube to reduce the headspace of the reaction. Cell-free protein synthesis was performed at 37°C for 6 h, and the reaction mixtures were kept on ice before fluorescence analysis. The production of sfGFP was estimated using fluorescence emission at 518 nm (excitation, 494 nm) on a microplate reader (Epoch 2 microplate reader, Agilent, CA, USA), with standard curves generated by purified sfGFP (50–350 µg/mL)[1].

In vitro transcription. *In vitro* transcription was performed using ScriptMax® Thermo T7 Transcription Kit (TOYOBO, Osaka, Japan) following the manufacturer's standard protocols. Briefly, DNA templates (0.7 µg) were added to the RNA transcription reaction mixtures (40 µL) and were incubated at 40℃ for 4 h. The RNA transcripts were then purified using NucleoSpin® Gel and PCR Clean-up Kit (Macherey-Nagel, Düren, Germany) and the concentrations were measured by NanoDropTM2000c spectrophotometer (Thermo Fisher Scientific, MA, USA).

Cysteine desulfurase activity assays. Total sulfide was measured based on a previously published protocol[3]. Reactions were carried out anaerobically in a buffer containing HEPES-Na (pH 7.5; 50 mM), NaCl (0.1 M), SufE (3 µM), SufS (0.5 µM), and glycerol (5%, v/v). Pyridoxal 5′-phosphate was added to 10 µM, and reactions were initiated by dilution of an L-cysteine/DTT stock (40 mM) to a 1 mM final concentration of each in a total reaction volume of 1 mL. Reactions were allowed to proceed for 15 min at 37℃ and then were quenched by mixing 100 µL of the reaction with 100 µL of N, N-dimethyl-p-phenylenediamine (17 mM), and $FeCl_3$ (22.2 mM) in HCl (6 M). Subsequently, the quenched reactions were incubated for 20 min, leading to the formation of methylene blue. Precipitated protein was removed by 30 s centrifugation at 20,000 × g, and methylene blue was measured with a UV-Vis scanning at $\lambda_{670\ nm}$ on a microplate reader (Epoch 2 microplate reader, Agilent, CA, USA).

Cytochrome C reduction optical assays. Oxidized ferredoxin is reduced enzymatically by spinach ferredoxin-$NADP^+$ reductase (Sigma-Aldrich, MO, USA) using NADPH. Reduced ferredoxin was used to reduce cytochrome C from the equine heart (Sigma-Aldrich, MO, USA) in a non-enzymatic manner. The formation of reduced cytochrome C is monitored by the time-dependent increase in $\lambda_{550\ nm}$. The reaction mixtures (300 µL) contained HEPES-Na (pH 8.1; 50 mM), NaCl (0.1 M), glycerol (5%, v/v), NADPH (1 mM), cytochrome C (0.1 mM), ferredoxin-$NADP^+$ reductase (0.25 µg/mL; Sigma-Aldrich, MO, USA), and reconstituted holo-Fd. The holo-Fd was diluted and assayed at concentrations ranging from 0.5 to 4.0 µM. The reaction was initiated by the addition of holo-Fd. Under aerobic conditions, assays were performed in an optical 96-well microplate at 30℃ for 25 min with a UV-Vis scanning at $\lambda_{550\ nm}$ every 5 s on a microplate reader (EnSpire®, PerkinElmer, MA, USA). All enzyme assays were conducted in duplicates or triplicates as indicated in the figures.

Time-course [4Fe-4S] cluster degradation under aerobic conditions. The mature $SufBC_2D$ complex produced under anaerobic conditions was incubated under aerobic conditions with and without the O_2-scavenging enzyme cascade. The reaction mixture contained HEPES-Na (pH 7.6; 50 mM), NaCl (100 mM), $MgCl_2$ (5 mM), dithiothreitol (1 mM), sodium formate (10 mM), NADH (0.1 mM), FAD (20 µM), formate dehydrogenase (0.05 mg/mL), flavin reductase (0.05 mg/mL), catalase (0.01 mg/mL), mature $SufBC_2D$ complex (10 µM), and glycerol (5%, v/v). The reaction mixture was assembled to a final volume of 200 µL in the 300 µL PCR tubes under anaerobic conditions, and the reaction was initiated by opening the lid under aerobic conditions. The reaction mixture was incubated at 37℃ for 0, 30, 60, and 90 min. Time-course [4Fe-4S] cluster degradation ($\lambda_{420\ nm}$) in the reaction mixtures was monitored using an Epoch 2 microplate reader (Agilent, CA, USA) under anaerobic conditions.

Aconitase A and isocitrate dehydrogenase-coupled activity assays. Aconitase activity was assayed using the procedure previously described[4] by monitoring the formation of NADPH through the increase in $\lambda_{340\ nm}$. The reaction mixture (100 µL) contained HEPES-K (pH 7.6; 50 mM), $MnCl_2$ (0.5 mM), citrate (10 mM), $NADP^+$ (1 mM), 2-mercaptoethanol (0.1%, v/v), and isocitrate dehydrogenase (IDH) (0.2 U; Sigma-Aldrich, MO, USA) with different concentrations of recombinant holo-AcnA. Note that the stock solution for the citrate (1 M) was adjusted to pH 7.6 in HEPES-K buffer (50 mM) to prevent a pH shift in the reaction mixture. The reaction was initiated by the addition of holo-AcnA. Under anaerobic conditions, assays were performed in an optical 96-well microplate at 37°C for 10 min with a UV-Vis scanning at $\lambda_{340\ nm}$ every 10 s on a microplate reader (Epoch 2 microplate reader, Agilent, CA, USA). The $\lambda_{340\ nm}$ was later converted to NADPH concentration based on the standard curves derived from the different concentrations of NADPH. All enzyme assays were conducted in duplicates or triplicates as indicated in the figures. The concentration of reconstituted holo-AcnA was estimated using a standard curve generated by serial diluted holo-AcnA overexpressed and purified from *E. coli*. Concentration of AcnA in the enzyme assays were estimated from the band intensity in the SDS-PAGE by using AcnA purified from *E.coli* as a standard.

LC–MS analysis The completed reactions of the AcnA and IDH-coupled activity assays were collected and filtered through the Amicon® Ultra-0.5 centrifugal filter (cut-off: 3 kDa) (Merck KGaA, Darmstadt, Germany), and the flowthrough fractions were used for an LC-MS system

consisting of an Acquity UPLC plus system (Waters, Milford, MA, USA) connected to XEVO G2-XS QTof Mass Spectrometry (Waters, Milford, MA, USA). The citrate and alpha-ketoglutarate were separated and detected as described previously[5]. Briefly, the separation of underivatized carboxylates was achieved by injecting 5 µL of a sample onto an Atlantis Premier BEH C18 AX column (100×2.1 mm, 1.7 mm; Waters, Milford, MA, USA). The mobile phase was composed of (A) 10 mM ammonium formate in the pH 2.6 water and (B) 10 mM ammonium formate in 50/50 water/methanol (v/v) of pH 9.4 (pH of aqueous buffer before mixing with methanol). The mobile phase was delivered to the column at a flow rate of 0.35 mL/min with the following gradient: 0.0 min (0% B), 0.5 min (0% B), 3.0 min (70% B), 6.0 min (70% B), 7.0 min (0% B), and 10.0 min (0% B). The column was thermostated at 30°C. Analytes were ionized in an electrospray ion source operated in the negative mode. The source and gas parameters were set as follows: capillary 2.50 kV, source temperature 100 °C, cone gas 100 L/h, desolvation temperature 300°C, and desolvation gas 490 L/h.

Supporting Tables

Table S1. Calculation of dissolved oxygen in the PUREfrex reactions (in a separate Excel spreadsheet).

Table S2. Complete list of recombinant proteins, expression vectors, and primers for gene cloning used in this work.

Protein name	Gene name	Organism	Uniprot ID	Expression plasmid	Original plasmid	Molecular weight (kDa)	Forward primer sequence (5′ to 3′)	Reverse primer sequence (5′ to 3′)
Fd	*frd*	*Thermococcus profundus*	Q9HHD4	pET-23a(+)	pET-23a(+)	7.8	TAATACGACTCACTATA GGG	CAATCCGGATATAGTTCC TCC
SufA	*sufA*	*Escherichia coli*	P77667	pET-26b(+)	pUC57	14.6	ATGCATCACCACCACCA CCATTCACAAGATCCAG ATATGCACTCAGGAACA TTTAAT	CCAACTCAGCTTCCTTTC GGGCTTTAACGATATCG CGCTATTTGCC
SufB	*SufB*	*Escherichia coli*	P77522	pET-26b(+)	pUC57	56.0	ATGCATCACCACCACCA CCATTCACAAGATCCATC AAGGAATACAGAAGCTA CTG	CCAACTCAGCTTCCTTTC GGGCTTTAACGATATCG CGCTATTTGCC
SufC	*sufC*	*Escherichia coli*	P77499	pET-26b(+)	pUC57	29.9	ATGCATCACCACCACCA	CCAACTCAGCTTCCTTTC

							CCATTCACAAGATCCACT ATCAATAAAAGATTTAC ACGTAAGT	GGGCTTTAACGATATCG CGCTATTTGCC
SufD	*sufD*	*Escherichia coli*	P77689	pET-26b(+)	pUC57	48.1	ATGCATCACCACCACCA CCATTCACAAGATCCAG CAGGACTACCCAACAGT	CCAACTCAGCTTCCTTTC GGGCTTTAACGATATCG CGCTATTTGCC
SufE	*sufE*	*Escherichia coli*	P76194	pET-26b(+)	pUC57	17.1	ATGCATCACCACCACCA CCATTCACAAGATCCAG CTTTACTACCCGATAAAG AAAAG	CCAACTCAGCTTCCTTTC GGGCTTTAACGATATCG CGCTATTTGCC
SufS	*sufS*	*Escherichia coli*	P77444	pET-26b(+)	pUC57	45.7	ATGCATCACCACCACCA CCATTCACAAGATCCAA TATTTTCAGTTGATAAAG TAAGGGCT	CCAACTCAGCTTCCTTTC GGGCTTTAACGATATCG CGCTATTTGCC
AcnA	*acnA*	*Escherichia coli*	P25516	pET-23a(+)	ASKA clone	99.8	CACCATCTGGAAGTTCTG TTCCAGGGGCCCTCACA AGATCCATCGTCAACCCT ACGAGAA	CTTTGTTAGCAGCCGGAT TCATTATTTCAACATATT ACGAATGAC
FRE	*fre*	*Escherichia coli*	P0AEN1	pET-23a(+)	pET-23a(+)	27.5		
FDH	*fdh*	*Pseudomonas sp. (strain 101)*	P33160	pET-26b(+)	pET-26b(+)	45.4		
CAT	*katE*	*Escherichia coli*	P21179	pET-21d(+)	pET-21d(+)	84.1		

Expression vector	Forward primer sequence (5′ to 3′)	Reverse primer sequence (5′ to 3′)
pET-23a(+)	ATCCGGCTGCTAACAAAGC	TGGATCTTGTGAATGGTGGTG / GGGCCCCTGGAACAGAACTTCCAGATGGT GGTGGTGGTGATGCAT (to insert HRV 3C cutting site sequence between N-His6 tag and *acnA*)
pET-26b(+)	AAAGCCCGAAAGGAAGCTGAGTTGG	TGGATCTTGTGAATGGTGGTGGTGGTGATG CATCATATGTATATCTCCTTCTTAAAGTTA AACAAAATTATTTC

Supporting Figures

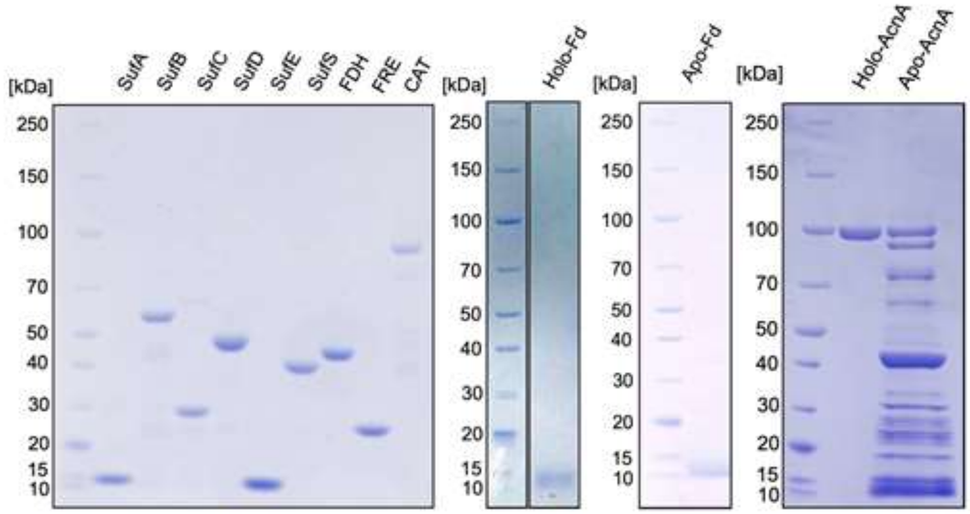

Figure S1. SDS-PAGE gel images of purified recombinant proteins and apo-Fd/AcnA synthesized by the PURE system. All recombinant proteins (SufABCDSE, FDH. FRE, CAT, Holo-Fd and Holo-AcnA) are overexpressed and purified from *E.coli*. Apo-Fd was expressed using PUREfrex2.0 and purified by boiling and centrifugation. Apo-AcnA was ran on the gel along with the protein components in the PUREfrex reaction mixture. We did not purify Apo-AcnA due to its application for the one-pot *de novo* synthesis of holo-AcnA.

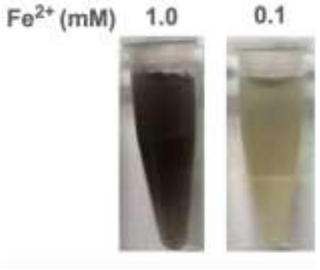

Figure S2. Images of the mature $SufBC_2D$ complex at different concentrations of $FeSO_4$

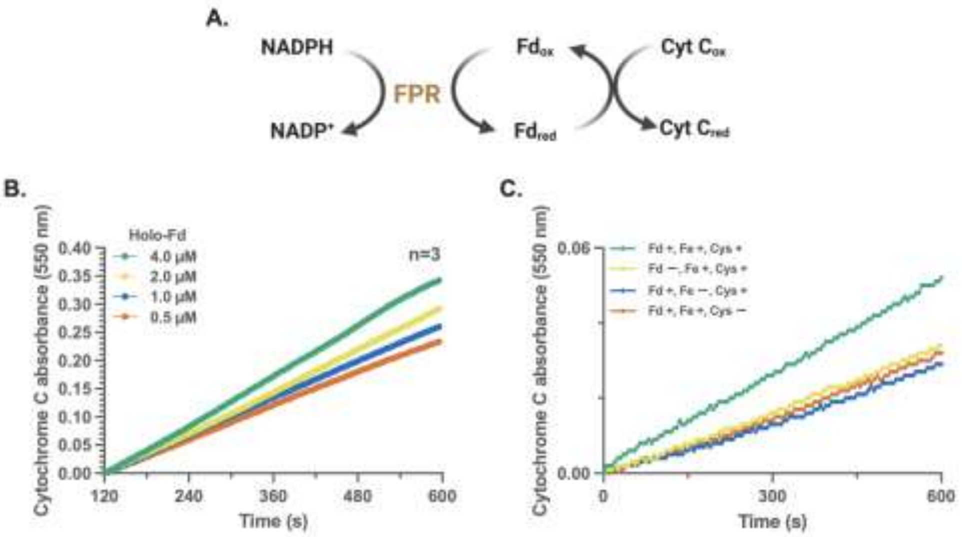

Figure S3. Standard curve to estimate the holo-Fd concentration using the cytochrome C-coupled optical assays. (A) Schematic diagram showing the enzyme cascade of the cytochrome C-coupled optical assays. **(B)** The activity of the recombinant holo-Fd was measured by the cytochrome C-coupled optical assays. The bars represent the standard error, and the dots represent the mean from three experimental replicates. **(C)** The activity of the anaerobically reconstituted Fd was measured by the cytochrome C-coupled optical assays. Apo-Fd was matured by the [4Fe-4S]-charged SufBC$_2$D complex with and without Fd, Fe^{2+}, and cysteine.

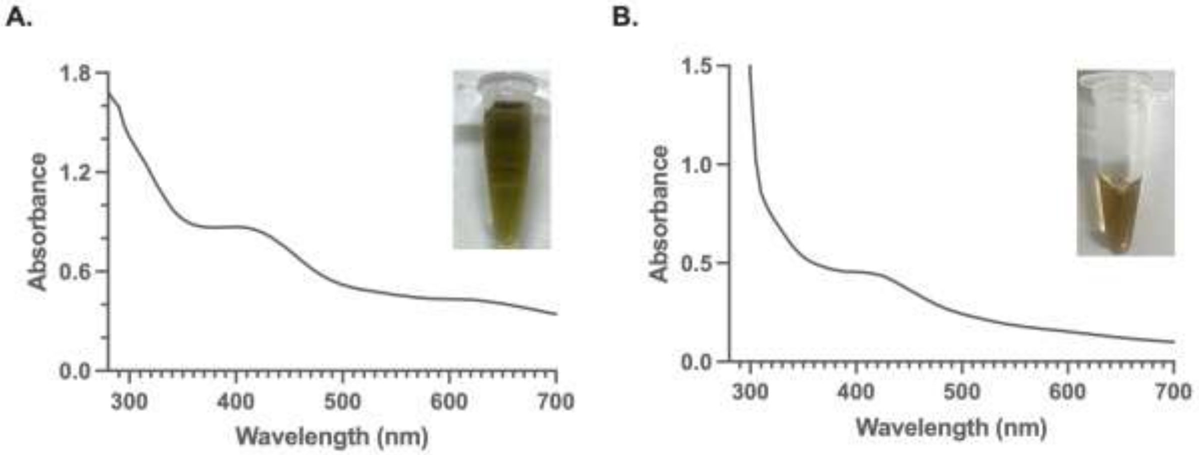

Figure S4. UV-Vis analysis of the recombinant proteins purified from the anaerobically cultivated *E. coli* BL21 cells. The two recombinant proteins **(A)** holo-Fd and **(B)** AcnA were purified in an anaerobic chamber under strictly anaerobic conditions. The UV-Vis analysis was

also performed in an anaerobic chamber. Visual image of the purified protein in a test tube are also presented.

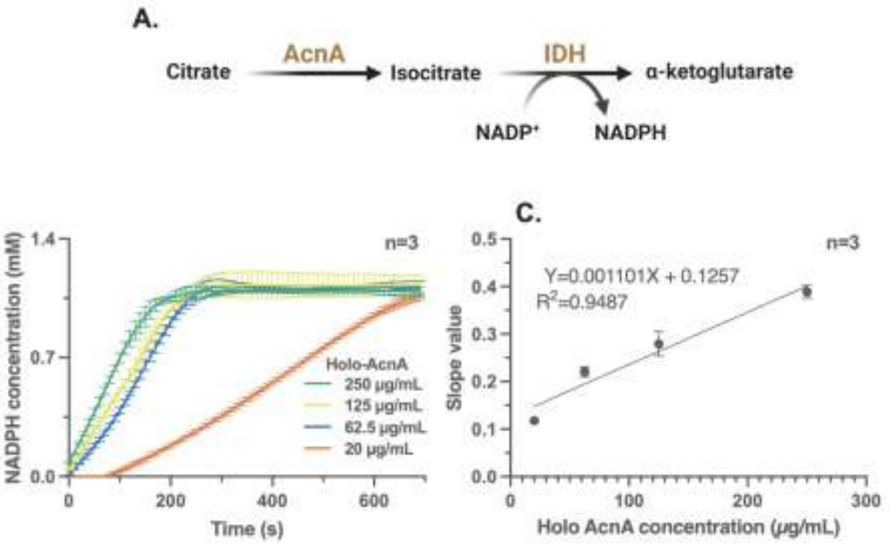

Figure S5. Standard curve to estimate the holo-AcnA concentration using the aconitase A and isocitrate dehydrogenase-coupled activity assays. (A) Schematic diagram showing the enzyme cascade of the aconitase A and isocitrate dehydrogenase-coupled activity assays. **(B)** The activity of the recombinant holo-AcnA purified from *E. coli* was measured by the aconitase A and isocitrate dehydrogenase-coupled activity assays. **(C)** Standard curves of holo-AcnA derived from the slope value (calculated from the linear range (100–130 s)) of the activity measured in (B). The bars represent the standard error and the dots represent the mean from three experimental replicates.

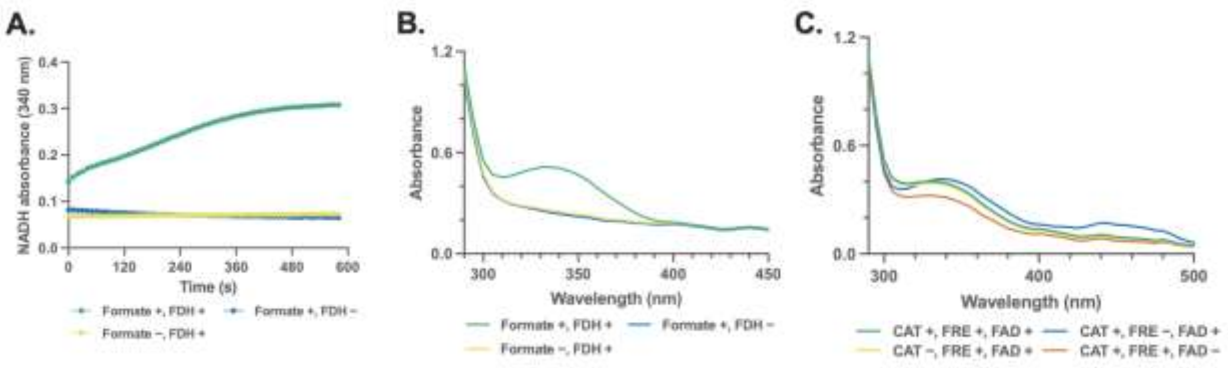

Figure S6. NADH and $FADH_2$ production using formate dehydrogenase and the O_2-scavenging system, respectively. (A) The production of NADH was monitored for 10 min using the absorbance at $\lambda_{340\ nm}$ in formate and formate dehydrogenase (FDH) supplemented PUREfrex buffer. **(B)** Reaction mixture subjected to UV-Vis analysis (300–450 nm) after 10 min incubation. **(C)** UV-Vis analysis of the PUREfrex buffer containing the formate, NADH and FDH, supplemented with/without flavin reductase (FRE), catalase (CAT) and FAD. The absorbances of NADH $\lambda_{340\ nm}$ and $FADH_2$ $\lambda_{445\ nm}$ were measured after 10 min incubation.

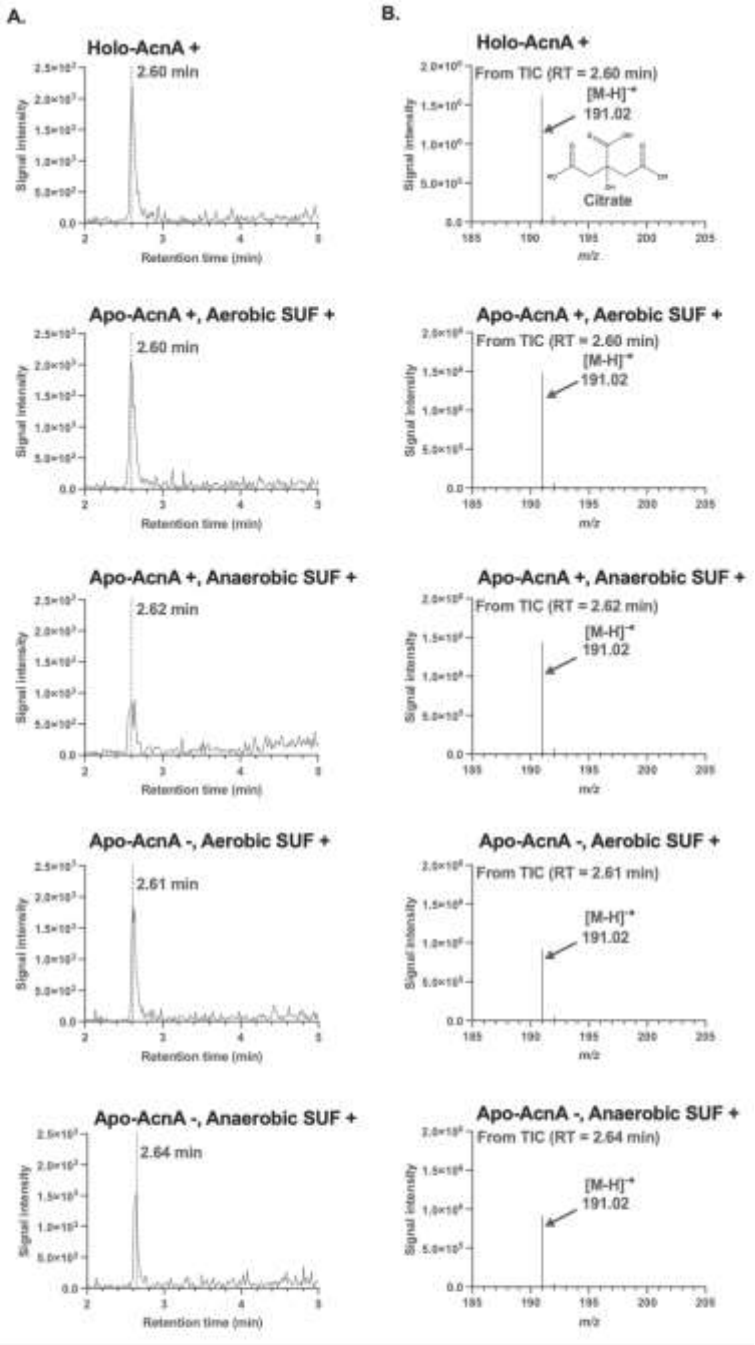

Figure S7. LC-MS spectra of citrate. (A) LC-ESI-MS extracted ion chromatograms and **(B)** MS spectrum of the completed reaction obtained from the aconitase A (AcnA) and isocitrate dehydrogenase-coupled assay. Apo-AcnA was produced in the PURE system and then the reactions were combined with the [4Fe-4S]-charged SUF under aerobic or anaerobic conditions. The experiment was conducted with and without Apo-AcnA. TIC, total ion current spectrum.

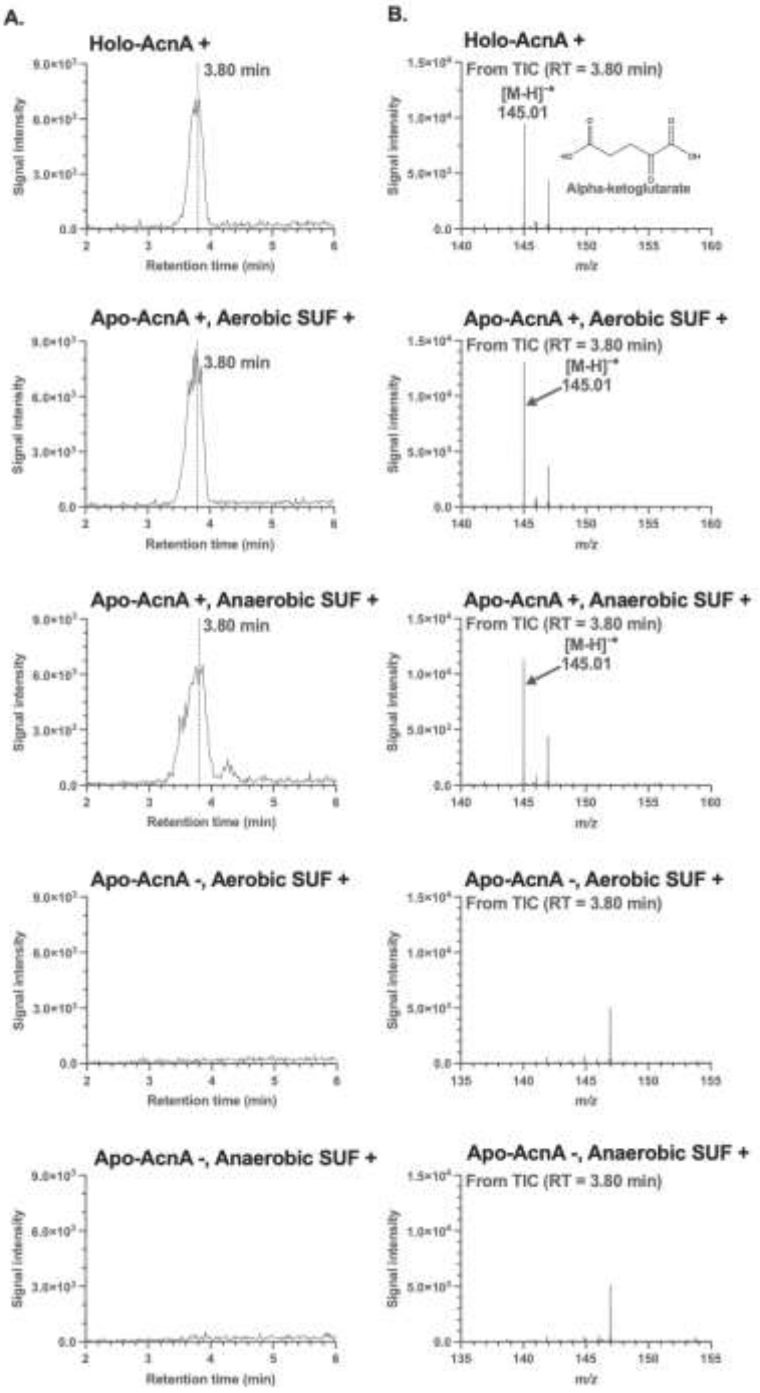

Figure S8. LC-MS spectra of alpha-ketoglutarate. (A) LC-ESI-MS extracted ion chromatograms and **(B)** MS spectrum of the completed reaction obtained from the aconitase A and isocitrate dehydrogenase-coupled assay. Apo-AcnA was produced in the PURE system and then the reactions were combined with the [4Fe-4S]-charged SUF under aerobic or anaerobic conditions. The experiment was conducted with and without Apo-AcnA. TIC, total ion current spectrum.

References

(1) Wang, P.-H.; Fujishima, K.; Berhanu, S.; Kuruma, Y.; Jia, T. Z.; Khusnutdinova, A. N.; Yakunin, A. F.; McGlynn, S. E. A bifunctional polyphosphate kinase driving the regeneration of nucleoside triphosphate and reconstituted cell-free protein synthesis. *ACS Synthetic Biology* **2020**, *9* (1), 36–42.

(2) Kuznetsova, E.; Proudfoot, M.; Gonzalez, C. F.; Brown, G.; Omelchenko, M. V.; Borozan, I.; Carmel, L.; Wolf, Y. I.; Mori, H.; Savchenko, A. V.; Arrowsmith, C. H.; Koonin, E. V.; Edwards, A. M.; Yakunin, A. F. Genome-wide analysis of substrate specificities of the *Escherichia Coli* haloacid dehalogenase-like phosphatase Family. *Journal of Biological Chemistry* **2006**, *281* (47), 36149–36161.

(3) Urbina, H. D.; Silberg, J. J.; Hoff, K. G.; Vickery, L. E. Transfer of sulfur from IscS to IscU during Fe/S cluster assembly. *Journal of Biological Chemistry* **2001**, *276* (48), 44521–44526.

(4) Gardner, P. R.; Fridovich, I. Inactivation-reactivation of aconitase in *Escherichia Coli*. A sensitive measure of superoxide radical. *Journal of Biological Chemistry* **1992**, *267* (13), 8757–8763.

(5) Hodek, O.; Argemi-Muntadas, L.; Khan, A.; Moritz, T. Mixed-mode chromatography-mass spectrometry enables targeted and untargeted screening of carboxylic acids in biological samples. *Analytical Methods* **2022**, *14* (10), 1015–1022.
